# The Beneficial Effects of Soluble Silicon Fertilizer in *Dendrobium* Orchids: Silicon-Augmented Resistance against Damage by Insect Pests and Fungal Pathogens

**DOI:** 10.3390/insects15050323

**Published:** 2024-05-01

**Authors:** Joanna Bloese, Russell Galanti, Ryan Porter, Tiani Know

**Affiliations:** College of Tropical Agriculture and Human Resources, University of Hawaii at Manoa, Hilo, HI 96720, USA; rgalanti@hawaii.edu (R.G.);

**Keywords:** silicon, *Thrips palmi*, *Frankliniella occidentalis*, dendrobium, fertilizer, *Fusarium* spp.

## Abstract

**Simple Summary:**

This study investigated the effectiveness of soluble silicon fertilizer (SiO_2_) in protecting dendrobium orchids from thrips pest complexes and fungal pathogens to enhance yield and quality. Over a nine-month greenhouse trial in Hawaii, three dendrobium varieties (‘Uniwai Supreme’, ‘Uniwai Mist’, and ‘Uniwai Royale’) received alternating weekly applications of soluble silicon fertilizer (50 mg Si/plant/application) via either soil drench and/or foliar methods. The primary objectives were to assess the impact of soluble silicon fertilizer on marketable yield, spray length, thrips and fungal damage, and plant vigor. The results revealed significant benefits of silicon fertilization across all varieties. ‘Uniwai Supreme’ exhibited the most substantial increase in marketable yield (73.0%) and ‘Uniwai Mist’ had the least increase in marketable yield, with a 50.6% increase compared to untreated controls. These findings have practical implications for orchid cultivation and pest management practices, demonstrating the potential of soluble silicon to improve orchid health and productivity by reducing pest damage. By mitigating the impact of thrips and fungal pathogens, silicon fertilization can contribute to more sustainable orchid production methods. Ultimately this research supports the adoption of silicon supplementation as a strategy to enhance Integrated Pest Management (IPM) tactics and promote environmentally friendly agriculture.

**Abstract:**

The effects of soluble silicon fertilization on monocots and dicots have been widely studied. However, little is known regarding its effects on protecting epiphytes against insect and fungal pests. The efficacy of silicon fertilizer to reduce damage by thrips pest complexes, namely: *Thrips palmi* Karny*, Frankliniella occidentalis* Pergande, *Chaetanaphothrips orchidii* Moulton, and *Chaetanaphothrips signipennis* Bagnall (Thysanoptera: Thripidae), and the fungal pathogens: *Botrytis cinerea* Persoon (Helotiales: Sclerotiniaceae) and *Fusarium* spp. Link (Hypocreales: Nectriaceae) was examined during a nine-month greenhouse trial in Hawaii. The trial assessed yield, quality, and pest damage across three common varieties of dendrobiums. All replicates received additional soluble silicon fertilizer applications alternating weekly between soil drench and foliar (50 mg Si/plant) applications. Yield, quality, and spray length, pest damage, plant vigor, SPAD, and leaf temperature were measured. Data were analyzed using a generalized linear model (glm) with repeated measures followed by post-hoc pair-wise comparisons in R, version 4.3.1. Treatment effects were significant at *p* < 0.001 for the majority of the explanatory variables including: marketable yield, spray length, thrips damage, and fungal damage. Overall, the lavender variety (‘Uniwai Supreme’) benefited the most from silicon applications with a 73.0% increase in marketable yield, compared to the white variety (‘Uniwai Mist’), which had an increase of 50.6% marketable sprays in contrast to its untreated control. Si benefits conferred to the purple variety (‘Uniwai Royale’) were intermediate to the lavender and white varieties. Although the magnitude of Si benefits varied among the varieties, all dendrobium varieties significantly benefited from silicon fertilization.

## 1. Introduction 

### 1.1. Dendrobium Production in Hawaii

Dendrobium (*Dendrobium* spp., (Swartz), Asperagales: Orchidaceae) production has a rich historical and cultural significance in Hawaii. Dendrobiums are commonly used in lei for celebrations, ceremonies, and hula. The island of Hawaii has many diverse climates that have led to an explosion of varied orchid types, species, and hybrids; thus, the majority of orchid production occurs on Hawaii island, and it has been endearingly nicknamed ‘The Orchid Isle’. The first statistical record of dendrobium production in Hawaii was published in 1969 by the Hawaii Department of Agriculture (HDOA), with 228,000 cut sprays (orchid inflorescence, that consists of a peduncle, the stalk of the inflorescence in which multiple blooms arise; ranges in length) sold for a wholesale value of USD 50,000 [1]. Historically, dendrobium production was one of the fastest growing agricultural industries in Hawaii [1]. In 2020, cut and potted orchids were valued at USD 12.2 million in Hawaii and dendrobium orchid sales alone were valued at USD 3.16 million [2]. This is a slight decrease from 2018 production values (USD 14 million for cut and potted orchids) due in large part to the 2018 Kilauea lava eruption and the negative impacts of the COVID-19 pandemic [3,4,5]. Damage to farmers from the Fissure 8 lava flow totaled USD 27.9 million in farm losses, with USD 13.3 million in losses from floriculture and the nursery industry, namely orchid production [3]. Orchid production in Hawaii is in the process of rebuilding from these events. Nationally, the industry continues to grow from 137 producers in 2018 to 175 producers in 2019 [6]. The total orchid wholesale value for 2019 was USD 305 million nationally, and among indoor houseplants potted orchids account for one-third of the total wholesale value of all potted flowering crops in the U.S. [6]. 

### 1.2. Pests of Dendrobiums

Hawaii orchid growers battle a wide range of insect and pathogen pests. The year-round growing season makes pest management especially challenging. Thrips are the most common insect pest of orchid flowers in Hawaii. There are over ten common thrips species and four fungal pathogens of dendrobium alone (Table 1). Thrips damage terminal bud growth leaving it stunted and deformed. At lower density levels damage can occur as scarring on the petals [7]. In severe infestations, thrips can damage every bloom produced in infested greenhouses [8], and destroy newly formed sprays, rendering yield to essentially zero. Without the proper management of thrips, dendrobium production would not remain profitable. Surveys conducted by the University of Hawaii and HDOA have identified western flower thrips (*Frankliniella occidentalis* Pergande (Thysanoptera: Thripidae)) and melon thrips (*Thrips palmi* Karny (Thysanoptera: Thripidae)) as the two most prevalent species of thrips infesting dendrobium [9]. These two invasive species were introduced to Hawaii in 1955 and 1982, respectively [10,11]. Populations can grow rapidly as they have a relatively short life cycle and can reproduce both sexually and asexually. Hawaii’s tropical climates allow pest populations to remain at damaging thresholds [12] year-round as temperatures do not significantly drop during winter months [13], highlighting the importance of sustainable pest management strategies. Both species of thrips are polyphagous, and their host range includes at least 50 different plant species [11,14,15,16,17]. Both species are vectors for several economically significant plant viruses, including at least six known tospoviruses specifically transmitted by *T. palmi* [18]. *Thrips palmi* is still classified as a federal quarantine action pest, which means there is a zero-tolerance level for orchids exported from Hawaii to the continental United States and other un-infested areas around the world. Entire flower shipments are rejected by inspection officials as a result of *T. palmi* and *F. occidentalis* infestations [19,20]. Due to these strict export regulations, dendrobium growers employ significant efforts to control pests. The international economic importance of *T. palmi* and *F. occidentalis* necessitates the development of sustainable management.

Other economically important pests include a range of fungal pathogens (Table 1). Major economically important pathogens of dendrobium orchids include gray mold (*Botrytis cinerea* (Persoon) Helotiales: Sclerotiniaceae) and fusarium (*Fusarium* spp. (Link) Hypocreales: Nectriaceae) [1,21,22]. Gray mold causes dark necrotic flecks on flowers, although other fungal species also incite necrotic flecking that resembles gray mold infections [21,23]. Fusarium has been reported as a pathogen in dendrobium in Hawaii and Asia, and causes necrotic leaf spots that can become severe blights. Fusarium also incites stem, pseudobulb, and root rot symptoms [22]. Humidity and airflow play a large role in fungal pathogens in dendrobium production and are the greatest environmental factors in promoting fungal problems for both the potted and cut dendrobium industry in Hawaii. 

### 1.3. Current Management Challenges

Dendrobium growers face a multitude of issues on the farm including an ever-shorter list of chemical control options for pests and a growing demand in the consumer marketplace for reduced pesticide use. Growers understand that the sole use of chemicals to control insect pests is not a sustainable long-term solution [24]. Currently, growers are battling issues with pesticide-resistant populations that have the ability to decimate entire nurseries, which include: *T. palmi*, *F. occidentalis*, *Chaetanaphothrips orchidii* (Moulton) (anthurium thrips), and *Chaetanaphothrips signipennis* (Bagnall) (banana rust thrips). Thrips are especially difficult to control due to their short life cycle, parthenogenetic reproduction, and their preference of hiding deep inside the blossom and petals, making contact with pesticides challenging [25]. Additionally, thrips pupae evade treatment as they are usually found in the soil and growing media, requiring carefully timed insecticide applications occurring in succession [26]. Thus, growers have come to heavily rely on a short list of systemic insecticides. 

*Fusarium* spp. can also be difficult to control if control measures are implemented too late in the disease cycle. Many available fungicides are prophylactic and there remain few therapeutic chemical options registered in Hawaii. Although growers often employ integrated tactics to control thrips and fungal pathogens, they rely heavily on the use of chemical pesticides. 

In 2000, a growers’ perception survey, conducted by the College of Tropical Agriculture and Human Resources (CTAHR) at the University of Hawaii at Manoa, found that 23.8% of growers reported that the average number of pesticide applications (excluding fungicides) made per year for growers of cut and potted orchids was 25.8 and 29.3 applications/year respectively [27]. Organophosphates and pyrethroids were the most common types of pesticides applied [27]. These high application rates reflect the industry’s reliance on costly chemical options.

The cost of agricultural inputs in Hawaii is further exacerbated by increases in shipping costs, most notably during the summer of 2020 due to the economic strain of the COVID-19 pandemic. The Public Utilities Commission approved a 46% rate increase for Hawaii’s only regulated inter-island cargo shipping company, Young Brothers [28]. 

Grower dependency on chemical pest control in dendrobium production in Hawaii generated the need for sustainable and economical alternative management options. Soluble silicon (Si) fertilizer is one input that has received attention for its potential use in controlling both biotic and abiotic stressors. A wealth of scientific evidence demonstrating the benefits of Si against a range of various environmental stresses has been established over the past several decades [29]; however, little research has been conducted on the effects of Si on epiphytic plants such as dendrobium. 

### 1.4. Silicon-Mediated Resistance

Si is a non-essential nutrient that is observed in substantial concentrations in plant tissues, and is linked to plant defense against insects and pathogens [30,31]. Silicon accumulation varies in plants and plants have previously been categorized by the percent of Si in dry shoot weight of the plant [32]. Si content can vary based on plant species and is also dependent on the application method, soil properties, Si source, and Si amount. Some orchid species were reported to absorb and show increased tissue Si concentrations in response to increased Si fertilization [33] and *Phaelanopsis* orchids are considered Si accumulators [34]. With the discovery of Si transporters, it has been suggested that Si accumulation should be categorized by the presence or absence of aquaporins [35]. No work was found on the presence of aquaporins in dendrobium. 

The understanding of how Si confers beneficial effects against insect pests and fungal pathogens has evolved over the last several years. Si was originally thought to provide primarily a physical defense, called the mechanical barrier hypothesis [36]. More recently, the mechanical barrier hypothesis has been challenged, and other mechanisms have been suggested, including defense priming [37,38,39,40], effector-triggered immunity [41], and the apoplastic obstruction hypothesis [42]. 

While there is limited available literature on Si effects in dendrobium [33]*,* the effects on thrips and fungi have been studied in other plant species. For example, Si has been shown to induce defense-related enzymes [43], induce photochemical efficiency, and adjust mineral uptake [44] in rice (*Oryza sativa* (L.) Poales: Poaceae) that help resist rice blast disease (*Magnaporthe oryzae*). Silicon was also found to positively affect sorghum (*Sorghum bicolor*) infected with anthracnose (*Colletotrichum sublineolum*) [45]. Additionally, Si has been proven to suppress *Fusarium* spp. in banana (*Musa acuminata*), tomato (*Solanum lycopersicum*), and potato (*Solanum tuberosum*) [46,47,48]. Not all studies showed efficacy against fungal pathogens in plants, especially necrotrophs [42], such as *Botrytis*. Previous studies have also examined thrips management using Si treatments with varied results. 

Several studies found that Si applications led to decreased thrips counts and lesions due to an increased mortality of nymphs in tomato [49] and eggplant (*Solanum melogena*) [50]. Similarly, reductions in thrips populations occurred after treatment with Si in groundnut (*Arachis hypogea*) [51]. However, in pepper plants (*Capsicum annuum*), researchers found that Si applications had no significant effect on thrips populations [52]. Likewise, Si applications did not affect thrips populations in sugar cane (*Saccharum* spp.), but did affect stalk borer (*Eldana saccharina*), indicating that Si-mediated resistance may be more developed in different plant tissues [53]. Mantovani et al. [34] found a net positive benefit of Si in dendrobium growth, with concentrations of 27 and 16 mmol L^−1^ from potassium silicate and monosilicic acid sources, respectively. To date, no studies have been published exploring the effects of Si against thrips pests and fungal pathogens in dendrobium production.

### 1.5. Objectives

The purpose of this study was to evaluate the effects of soluble silicon dioxide (SiO_2_) on naturally occurring thrips and fungus populations on three popular dendrobium cultivars (‘Uniwai Mist’, ‘Uniwai Supreme’, and ‘Uniwai Royale’) in a greenhouse production setting. The nature of the experimental design allowed for the evaluation of the short and long-term effects of SiO_2_ on the two most common dendrobium pests (thrips and fungus) in Hawaii, and examination of the potential differences in response to Si in three popular varieties.

## 2. Materials and Methods

### 2.1. Experiment Design

An experiment investigating the effects of soluble silicon fertilizer on three popular varieties of dendrobium orchids was conducted at Waiakea Research Station (19.644–155.080), Hilo, Hawaii from 1 August 2020 to 30 May 2021 in a greenhouse (Conely’s Greenhouse Manufacturing and Sales, Montclair, CA, USA) with 30% shade. Treatments were arranged in a split plot design with main plots arranged in an RCBD, consisting of a negative control versus soluble silicon dioxide fertilizer (Mainstay Si^®^, Redox (SiO_2_)) and three popular cultivars as sub plots, totaling 6 blocks. Subplots consisted of three varieties of dendrobiums: white (*Dendrobium* cv ‘Uniwai Mist’), lavender (*Dendrobium* cv ‘Uniwai Supreme’), and purple (*Dendrobium* cv ‘Uniwai Royale’). Dendrobium orchids were 3 years mature at the onset of the experiment. Si was applied at a rate slightly higher than the recommended drench label rate of 30.59 L/ha weekly, switching between foliar and drench applications. This rate was calculated to deliver 50 mg Si to each plant at each application [54]. We applied the fertilizer solution using a one-liter hand pump sprayer with a TeeJet^®^ flat fan nozzle, size 8004, for foliar applications to the point of full coverage, and applied 200 mL of solution to the root zone for drench applications via a 250 mL beaker vessel by hand. Overhead sprinklers irrigated plants for 3 min twice daily, using municipal water, and RainBird^®^ Micro-Quick 0.038-inch diameter sprinkler nozzles. 

Dendrobiums were grown in blue rock medium in plastic bags. All experimental units received Nutricote^®^ 14-14-14 (American Horticultural Supply, Inc.©, Oxnard, CA, USA), a controlled-release fertilizer at a rate of 336/kg/ha/year at the onset of the trial and 6 months into the trial, in keeping with conventional cultivation practices of dendrobium in Hawaii [1]. We relied on naturally occurring insect and fungal populations in the field. No pesticides were applied throughout the duration of the trial.

### 2.2. Data Collection

Data on several dependent variables were collected throughout the trial and can be categorized into the three categories outlined below. 

Harvest and quality: Sprays that had reached 67–75% maturity (meaning two thirds to three fourths of their lower blooms had fully opened and the top remaining 25–30% buds were unopened) were harvested every other week and yield and quality were assessed during the time of harvest [1]. Quality was characterized by: (i) percent damage to sprays, stems, blooms, and buds, and (ii) length of spray (cm). Sprays with less than 80% damage to blooms and stem were deemed salable. The remaining sprays were considered unmarketable and were considered ‘throw aways’. Harvest and quality data were collected for nine months between 9 September 2020 and 15 May 2021. Market prices are higher for longer sprays and an important consideration for grower profits. 

Insect and Disease Monitoring: Incidence of pest and disease was monitored weekly for five months between 25 September 2020 and 25 February 2021. Proportional thrips damage to blooms, sprays, buds, and stems was assessed. Scarring on flower sepals, petals, buds, and stems, as well as deformed and arrested spray development due to thrips damage was quantified on a percent damage scale from 0–100. No attempt to attribute damage to specific species was made as thrips damage on dendrobium presents similarly across all species. Additionally, pest counts were collected at the same time, however, thrips count is a less precise measure of thrips populations in the field, as many are small and hide in tight places of the orchid making them difficult to observe. To avoid disturbing the natural thrips populations in the greenhouse, we minimized the sampling through thrips removal. However, thrips were identified to species from representative field samples every 2 months and the populations consisted of *T. palmi*, *F. occidentalis*, *C. signipennis*, and *C. orchidii.*

Additionally, other insects were recorded through direct visual observation of both beneficial (*Orius tristicolor* White (Hempitera: Anthocoridae)) and pest species. Sampling was standardized by allowing observation to occur over a 30 s interval per plant and only one person collected this data for the duration of the trial.

Fungal pathogens were characterized by percent area of leaf affected by fungal legions, necrotic or blackened leaf spots, or percent area affected by dark necrotic flecks on the flowers*. Fusarium* sp. and *Botrytis* spp. were the only two fungal pathogens observed throughout the trial. Their symptoms were very distinct from each other and easily identified in the field. In addition, samples were collected, and pathogens were identified in laboratory via the microplate method.

From these measurements, we were able to analyze total disease, total thrips damage, and total beneficials in the analysis. 

Plant health and growth: Data were collected weekly on the number of new canes (dendrobium stems/stalks), and number of new sprays for nearly seven months, between 1 July 2020 and 22 January 2021. Data collection began one month prior to treatment applications to determine baseline plant vigor measurements. Soil Plant Analysis Development (SPAD) chlorophyll measurements and leaf temperature data were collected every 14 days between 30 August 2020 and 2 October 2020. The SPAD index was measured (portable chlorophyll meter; SPAD-502, Minolta, Japan) as it is one of the most commonly used diagnostic tools to measure crop nitrogen status in the field [55]. SPAD measurements were taken at a position two thirds from the leaf base on the newest mature leaf of the tallest cane. A total of three SPAD measurements per leaf were recorded and averaged [56]. Leaf temperature was measured using a Klein^®^ dual laser infrared thermometer at an optimal distance of 30.5 cm from the newest mature leaf. Three measurements per leaf were recorded and averaged. Leaf temperature was considered because it has been recognized as important for plant function and influences photosynthesis, respiration, and transpiration [56]. 

Vase Life: Harvested flowers were transported to Komohana Research and Extension Center (19.696–155.090), Hilo, Hawaii, where the stems were cut, and flowers were transferred to vases that were filled with room-temperature distilled water. The number of individual flowers on each sample inflorescence was counted. Flowers were kept in vases at an ambient temperature of 22.2 °C. Every five days vase water was replaced, and the number of individual flowers was counted on each sample inflorescence. Once the number of flowers reached half or less than half of the original number of flowers, that sample was discarded. Days to discard following harvest was recorded as the vase life measurement. Vase life data were collected later in the trial from 1 December 2020 to 1 June 2021. 

Silicon Accumulation: Six months into the experiment on 16 December 2020, we collected five mature leaves from each experimental unit, and dried leaves at 60 °C for 72 h in a Despatch^®^ model LBB2-27-1, 4800-watt oven, after which we sent them to the Agricultural Diagnostic Service Center (ADSC) at the University of Hawaii for analysis. Silicon content was determined gravimetrically in the plant tissue as the residue after acid digestion (Snyder 2001). 

Analysis: Statistical analyses were performed using R. 4.3.1 in RStudio [57,58]. Normally distributed data were analyzed using a R core anova function. Count data were analyzed using Generalized Linear Model in the glmm package [59] using a poisson distribution. Post-hoc pairwise comparisons were performed using Tukey’s HSD following ANOVA analysis and estimated marginal means (emmeans + contrast) following GLM [60].

## 3. Results

Results indicated a significant benefit of soluble silicon fertilizer (*p* < 0.001) among insect and disease, spray length, and marketable yield.

### 3.1. Harvest

**Figure 1:**
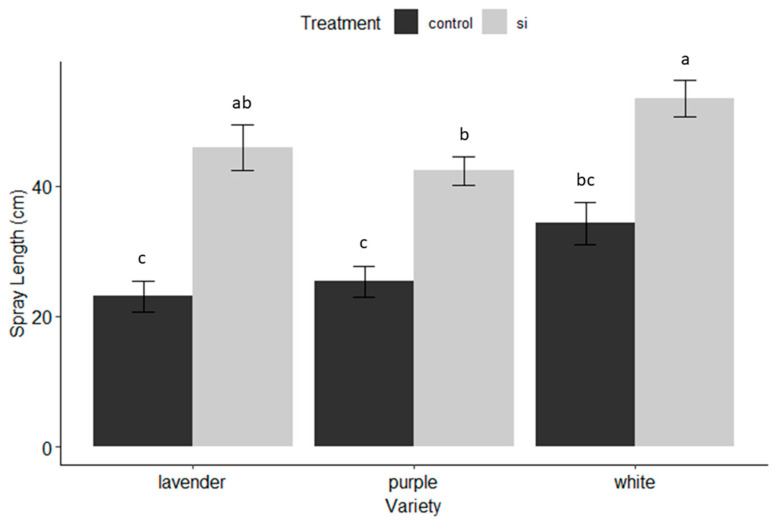
Spray length (cm) at harvest (67–75% mature blooms) by dendrobium cultivar and treatment (Si versus untreated control). Letters indicate significant differences between treatments according to pairwise comparisons between groups using R’s emmeans pairs() function.

*Spray length at harvest:* This experiment demonstrated that soluble silicon fertilizer had a significant influence on the spray length at harvest (F value = 69.208; df = 1, 189, *p* < 0.001) (Figure 1). The variety factor was also significant (F = 7.272; df = 2, 189, *p* < 0.001) with Si-treated white having the longest sprays (M = 53.4 cm ± 2.8), and was significantly longer than the Si-treated purple variety, which had the shortest sprays (M = 42.3 cm ± 2.1) among the treated varieties, neither being significantly different from the treated lavender in terms of spray length (Figure 1). However, the lavender variety demonstrated the largest benefit of Si in terms of spray length, with an average increase spray length of 22.9 cm ± 2.9 and purple benefiting the least in spray length from silicon fertilizer, with an average increase spray length of 16.9 cm ± 2.3 (Figure 1). 

**Figure 2:**
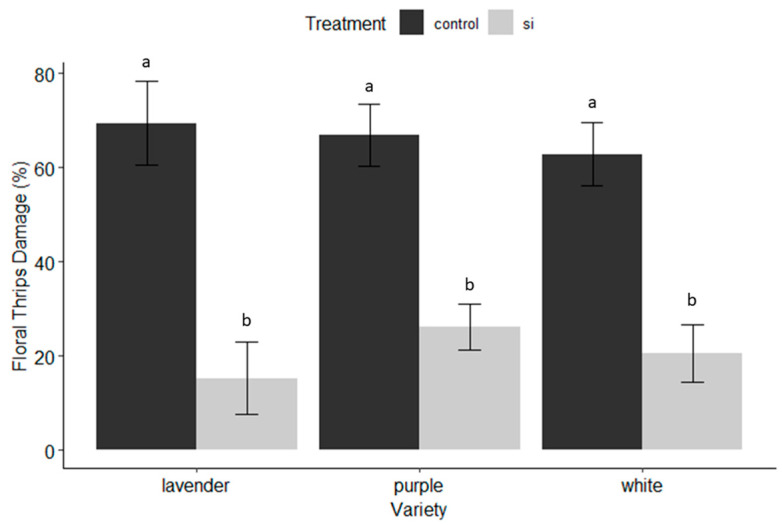
Proportion of thrips damage to blooms, sprays, buds, and stems, as well as deformed and arrested spray development due to thrips damage on a percent damage scale from 0–100 by dendrobium cultivar and treatment (Si versus untreated control). Letters indicate significant differences between treatments according to pairwise comparisons between groups using R’s emmeans pairs() function.

*Thrips damage on harvested sprays:* The amount of thrips damage observed at harvest was statistically significant between Si-treated and untreated control plants irrespective of variety (F = 64.5; df = 1, 189, *p* < 0.001). We observed the greatest amount of thrips damage in the control lavender variety and the least amount of thrips damage to the Si-treated lavender variety. Again, this demonstrated that the lavender variety benefited the greatest from the Si treatment, while the purple variety benefited the least. The average difference in percent thrips damage in Si- versus control-treated plants was 54.1% ± 8.3 and 40.6% ± 5.8, for lavender and purple varieties, respectively (Figure 2). 

**Figure 3:**
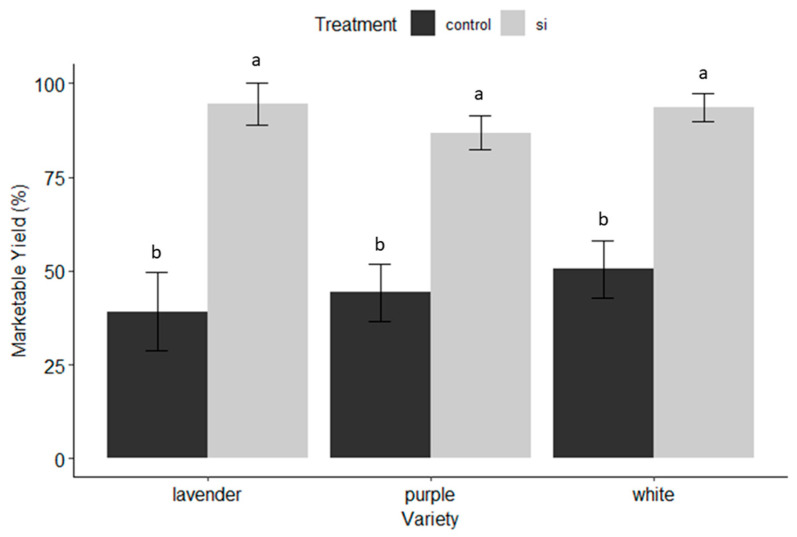
Percent marketable yield by dendrobium cultivar and treatment (Si versus untreated control). Marketable yield determined as percent of total sprays with less than 80% damage to blooms and stem and thus deemed salable. Letters indicate significant differences between treatments according to pairwise comparisons between groups using R’s emmeans pairs() function.

*Marketable yield:* The degree of thrips damage observed ocularly translated to marketable yield, and the same trend held true. Silicon effects on marketable yield were significant (F = 63.977; df = 1, 189, *p* < 0.001) while the variety term was not significant at α = 0.05. Lavender again benefited the greatest with a 55.3% increase in marketable yield, compared with white, which had an increase of 43.1% in marketable sprays, and lastly purple with the 42.7% increase in marketable sprays (Figure 3). 

### 3.2. Insect and Disease Monitoring

**Figure 4:**
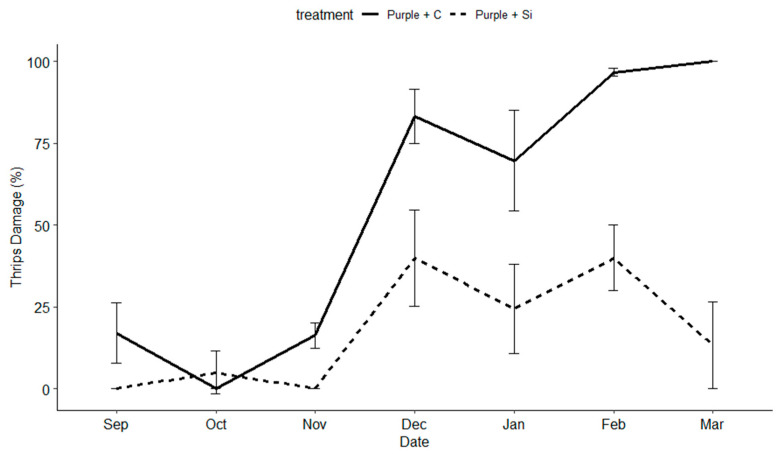
The percentage of thrips damage to blooms, sprays, buds, stems, and canes on the purple dendrobium variety (‘Uniwai Royale’) over the length of the trial from 1 September 2020 to 1 March 2021.

**Figure 5:**
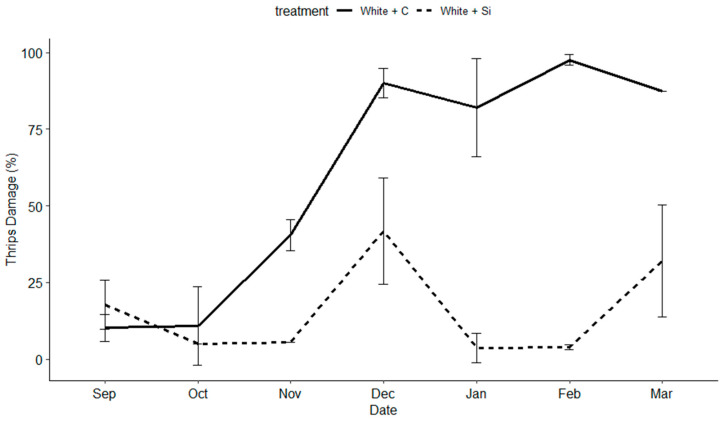
The percentage of thrips damage to blooms, sprays, buds, stems, and canes on the white dendrobium variety (‘Uniwai Mist’) over the length of the trial from 1 September 2020 to 1 March 2021.

**Figure 6:**
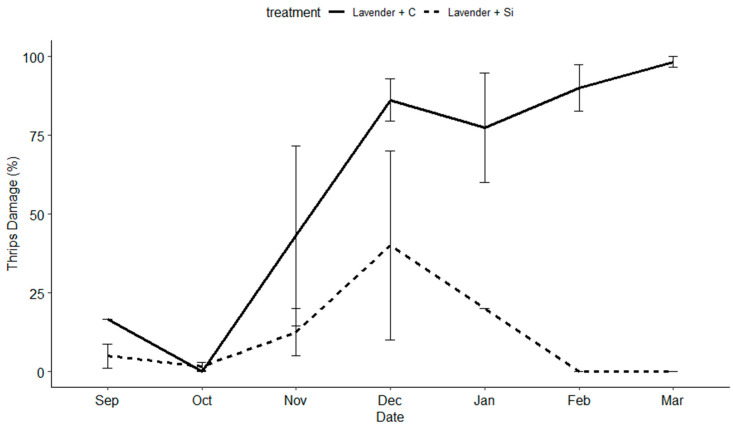
The percentage of thrips damage to blooms, sprays, buds, stems, and canes on the lavender dendrobium variety (‘Uniwai Supreme’) over the length of the trial from 1 September 2020 to 1 March 2021.

*Thrips damage:* Silicon fertilizer significantly lowered thrips damage (F = 147.223; df = 1, 175, *p* < 0.001) across all varieties. Variety was not a significant factor. However, date played a significant role (F = 31.677; df = 16, 175, *p* < 0.001) (Figure 4, Figure 5 and Figure 6). Differences among treatments were observed between six and eight weeks, becoming significant (α = 0.05) between eight and twelve weeks post the initial Si applications.

**Figure 7:**
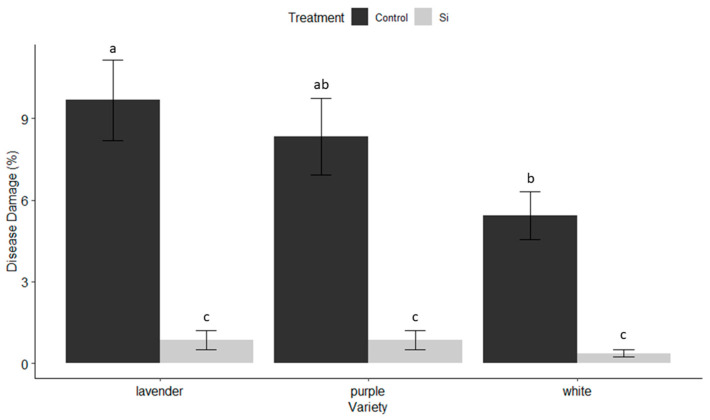
Percentage of leaf and flower area exhibiting disease symptoms (*Botrytis cinerea* and *Fusarium* spp.) by dendrobium cultivar and treatment (Si versus untreated control). Disease symptoms were characterized by percent area of leaf affected by fungal legions, necrotic or blackened leaf spots, and/or percent area showing dark necrotic flecks on the flowers per plant. Letters indicate significant differences between treatments according to pairwise comparisons between groups using R’s emmeans pairs() function.

*Fungal Damage:* The degree of infection by *B. cinerea* was minor over the length of the study. For this reason, we combined all fungal data into one data set. Significant differences were evidenced among the total disease observed throughout the life of the trial among treatment and variety factors (F = 88.341, df = 1, 188, *p* < 0.001; F = 3.469, df = 2, 188, *p* = 0.0322 respectively). We observed the greatest fungal infections on the untreated control lavender variety (M = 9.6% ± 1.5), which was significantly different from the untreated white variety, which exhibited the least amount of fungal pathogen damage; neither were significantly different from the untreated purple variety (Figure 7). The Si-treated white variety had the lowest infestation rate (M = 0.367% ± 0.13). Varieties with the greatest fungal damage benefited the most from the use of Si (Figure 7).

### 3.3. Plant Growth and Health

*Plant Vigor*: Plant vigor was determined by the sum of total sprays after seven months of collecting data weekly. There were no significant differences (α = 0.05) between Si-treated and untreated plants in terms of the number of sprays produced, although the total number of sprays produced was significant among varieties (F = 22.977, df = 2, 189, *p* < 0.001). Purple was the highest-yielding variety, followed by white and lavender (Table 2). The purple variety was the greatest-producing cultivar but overall benefited the least from Si applications, while the lavender variety was the lowest-producing variety and overall benefited the most from Si applications (Figure 1, Figure 2 and Figure 3 and Figure 7).

**Figure 8:**
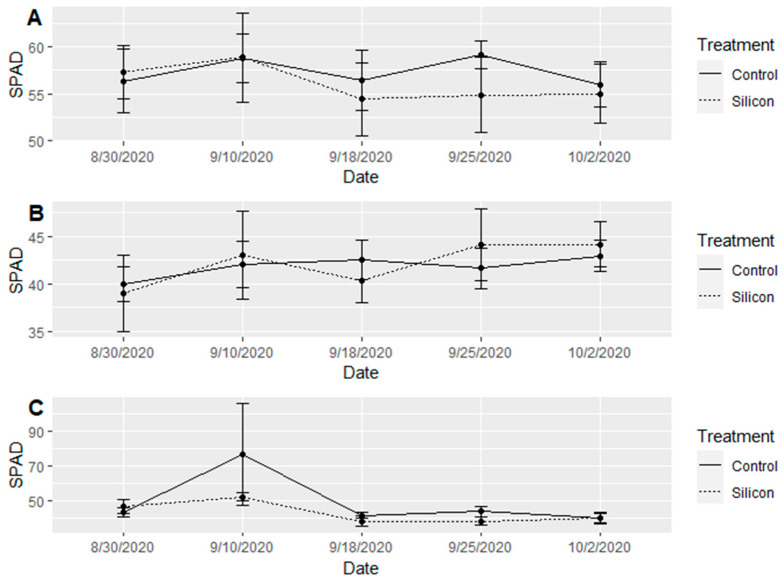
SPAD index (portable chlorophyll meter) measurements taken every 14 days between 30 August 2020 and 2 October 2020 across all varieties and treatment factors. (**A**) Mean SPAD readings (±SE) over time for Si-treated and untreated control lavender variety plants. (**B**) Mean SPAD readings (±SE) over time for Si-treated and untreated control white variety plants. (**C**) Mean SPAD readings (±SE) over time for Si-treated and untreated control purple variety plants. No significant differences were observed among treatments.

**Figure 9:**
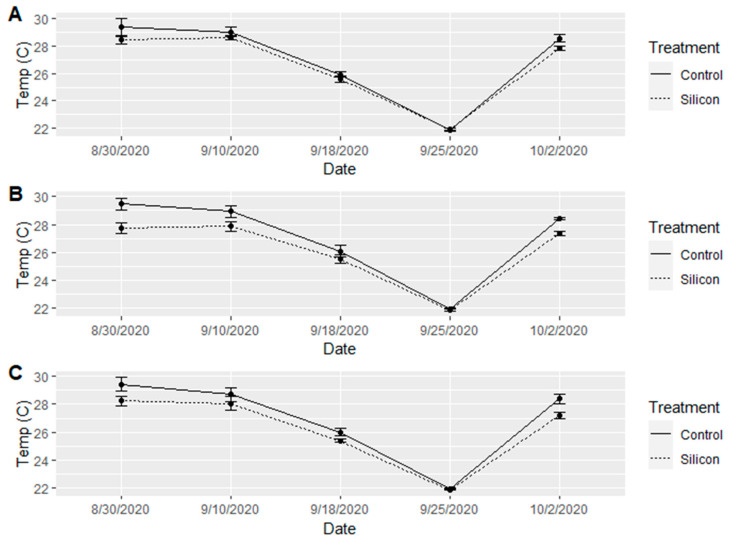
Mean leaf temperatures (°C) collected every 14 days between 30 August 2020 and 2 October 2020 across all varieties and treatment factors. (**A**) Mean leaf temperatures (±SE) for Si-treated and untreated control lavender variety plants. (**B**) Mean leaf temperatures (±SE) for Si-treated and untreated control white variety plants. (**C**) Mean leaf temperatures (±SE) for Si-treated and untreated control purple variety plants.

*SPAD and Temperature measurements*: There was no significant difference (α = 0.05) between treatment effects on SPAD readings (Figure 8); however, there were significant differences among the varieties in SPAD readings (F = 14.797, df = 2, 174, *p* < 0.001). The white variety consistently had the higher SPAD measurements, and the lavender variety consistently had the lowest reading. Leaf temperature was not significant (α = 0.05) between treatments, nor among varieties. It is important to note that the leaf temperature between treated and control plants was marginally significant at (α = 0.10, F = 2.91, df = 1, 174, *p* = 0.0897), and the Si-treated plants had a consistently lower leaf temperature for the majority of the five weeks we collected data; however, that difference ranged from 0.5–1 °C depending on variety (Figure 9). We see greater temperature differences among treatment groups at higher ambient temperatures and little to no difference among treatments below 23.5 °C.

### 3.4. Vase Life

A formal ANOVA could not be conducted due to the unbalanced nature of vase life data. When we began collecting vase life data four months into the trial, there were already high thrips densities in the greenhouse and untreated control varieties had very low yield, thus there were over 6.5 times the yield of Si-treated sprays versus control. On average, Si-treated sprays exhibited a longer vase life, days to discard (M = 25 days ± 7.2), than their control counterparts (M = 13 days ± 8.8), with no differences observed among varieties. Further research is needed to explore the potential benefit of Si on improving vase life.

### 3.5. Silicon Accumulation

**Figure 10:**
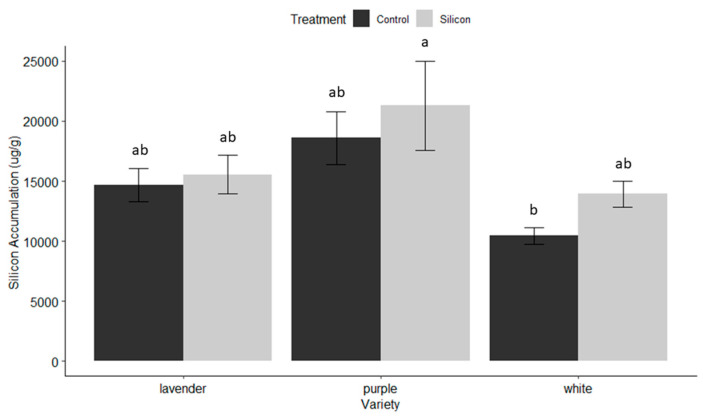
Silicon accumulation (μg/g) across all variety and treatment levels determined gravimetrically in the leaf plant tissue as the residue after acid digestion. Letters indicate no significant differences between Si-treated and untreated controls for any of the varieties according to Tukey’s HSD.

All Si-treated plants had slightly higher Si accumulation (μg/g) but none significantly higher than their untreated counterparts (Figure 10). 

## 4. Discussion

The benefits of soluble silicon fertilization in dendrobium production in Hawaii are evident throughout the results of this trial. On average, irrespective of variety, Si-treated dendrobium exhibited 53.4% less damage from thrips, and a 91% reduction in pathogen infection, which translated to varietal yield increases of 43.1–55.3%. These dramatic benefits were observed even though there was no significant increase in Si accumulation in treated versus untreated plants. The varieties that received the greatest benefit from Si (Figure 1, Figure 2 and Figure 3 and Figure 7) exhibited the least Si accumulation within its plant tissues (lavender variety, Figure 10), thus challenging our conventional understanding of the mechanisms of Si regarding crop protection.

The mechanisms by which Si provides protection against arthropod pests and disease is still largely unknown and varies by system. Historically, Si was believed to offer plant protection mainly by acting as a physical barrier to reduce insect feeding and deter disease infections. Silicon has been demonstrated to enhance phytoliths within plant tissues and change the shape and size of trichome bases on leaves [61]. Both aid in deterring arthropod feeding by herbivorous pests. One of the original theories developed from evidence that Si thickened the plant cell walls, making it more difficult for insects with piercing-sucking mouthparts to penetrate plant tissue, and/or wore down the mandibles of chewing insects, such as caterpillars. However, our Si accumulation data indicate that Si may work more as a cofactor or inducing factor that has a cascading effect and potentially changes gene expression and/or plant cell architecture (among other things) to provide plant protection, rather than on accumulation alone. Additionally, the dynamics of Si accumulation or sensitivity may vary in different tissues (Dr. Jon Suzuki 2023 personal communication). 

Recent evidence suggests that Si plays a crucial role in the Systemic Acquired Resistance (SAR) of plants [62]. SAR is understood to confer long-lasting protection against a broad spectrum of microorganisms. There is research to support the theory that indirect defenses by Si are mediated by herbivore-induced plant volatiles (HIPVs) released in response to insect feeding [31]. These HIPVs, modulated by the Jasmonic acid (JA) pathway, promote biological control by attracting predators and parasitoids of insect pests. Therefore, understanding the role of Si as part of both a physical barrier and as part of an induced-defense system is vital to knowing how to incorporate it as part of a larger IPM program. 

Our results show the promise of incorporating Si as part of an IPM program for dendrobium production in Hawaii and possibly other epiphytic cropping systems such as Anthuriums and Bromeliads. The incorporation of Si can be considered a ‘Bottom-Up’ tactic, defined as part of ecologically based methods that impact the tri-trophic interactions among crop, pest, and predator within agroecosystems to optimize IPM. Bottom-up management tactics often rely on strengthening natural plant defenses through nutrient management. Slow-growing plants, such as dendrobiums, require adequate time for the uptake and metabolism of fertilizers and other plant protection inputs. Understanding the effects of Si on invasive thrips pests is critical to the development and understanding of its complete benefits, offering a greater understanding of the biological trade-offs and practical insights into the relationships between plant nutrition, plant stress responses, pests, and diseases.

### 4.1. Role of Si in an IPM Program

There are many possibilities for successfully incorporating Si into an existing IPM program. These possibilities include: adding Si to media/soil as a supplement to growers’ current fertilizer regime, adding soluble Si to tank mixes during pesticide applications, and incorporating soluble Si in irrigation or fertigation systems during times of high pest pressure or likely abiotic stress. There is also the potential to incorporate Si applications into a pesticide resistance management program since it may offer plant protection with little to no selection pressure. To potentially utilize Si as part of a pesticide resistance management program, there are many questions that require further examination, the difficulty being that the answers to these questions may differ by cropping system. 

For Si to function as an effective part of a pesticide resistance management program, we would need to understand the following basic questions: (1) Does Si reduce pest population densities? (2) Does Si alter pest host selection? (3) Does Si alter plant tissues to make them a less nutritious host for herbivore pests? (4) Does Si make it more difficult for pests to penetrate plant tissue? (5) Does Si change the chemical ecology, specifically HIPVs, of the plant? (6) Are none of these factors altered (population density, host selection, nutrition, rigidity, and cell wall thickness) and damage is simply less visible on plants treated with Si?

Applied questions regarding application rates, methods (foliar versus drench), timing of application, and use in conjunction with pesticides also need to be considered when developing Si as a practical IPM tool. However, our results indicate that Si has the potential to positively impact the production of dendrobium, and possibly other epiphytic crops, in Hawaii.

### 4.2. Where and When Si Shines

Throughout the trial we observed that some varieties benefited more from Si than others. Although this difference was not statistically significant, this observed variation could be due to various factors. In our trial, the lavender variety experienced the most damage by thrips and fungal diseases, and also the greatest yield benefit from Si against both pests (Figure 2, Figure 3 and Figure 7). We believe the degree of benefit of Si is correlated to the degree of the stressor. It could also be that plant protection provided by Si is variety dependent and depends on the presence of certain Si transporter genes, along with other factors to allow for the uptake and transport of Si. The fact that Si has been shown to help with a variety of abiotic and biotic stressors in the field, including: insect pests, pathogens, heat and water stress, etc. [30,33,39,43,44,45,47,48] and that the benefits of Si have been variable across pest species, pest complexes, and cultivation systems [51,52], muddies not only our understanding of Si, but also how to best incorporate it into any IPM system. Within floriculture and nursery industries, the diversity of plant species and cultivars within species is vast and it is common, due to traditional breeding, for some cultivars to be highly resistant to certain pests, disease, and other abiotic factors, and susceptible to other like factors. It may be that Si will most benefit each cultivar in the area in which it is most susceptible. In this scenario, researchers must evaluate each cultivar’s susceptibility to any given pest, and collect data on the proper parameters to capture the potential benefit of Si. 

Additionally, the timing and length of application played an important role in the success of this trial. We did not begin to see the beneficial effects of Si until weeks 10–12. If we had concluded the trail after 2 months, and were only interested in short-term effects, we would not have necessarily seen positive results and missed the delayed benefits of Si. In the case of this trial, Si had a cumulative effect over time, providing plant protection against four common thrips pests and two classes of common fungal pathogens. However, we observed a diminishing return on investment after 5 months of weekly applications depending on the variety (Figure 4, Figure 5 and Figure 6). No pesticides were applied during this trial and future research should examine the use of Si in conjunction with pesticides and its possible value as a supplement to pesticide programs.

## 5. Summary

While it is clear that dendrobium orchids benefit from Si applications, the timing, rate, application method, frequency of application, and context in which it is utilized will require further development by researchers. The variability of Si to confer benefits against various pests and diseases across different cultivars further compounds the difficulty in determining the proper application factors described above. Understanding the short-term and long-term effects of Si and its ability to be used prophylactically and/or therapeutically will give us a better understanding of how to sustainably utilize this element in various stages of plant growth and cropping systems. 

## Figures and Tables

**Table 1:** List of the most common thrips and fungal pests of dendrobium orchids in Hawaii.

Scientific Name	Common Name	Categorization
*Chaetanaphothrips orchidii* *	Anthurium thrips	Thrips
*Frankliniella occidentalis* *	western flower thrips	Thrips
*Frankliniella shultzei*	yellow flower thrips	Thrips
*Haplothrips gowdeyi*	black flower thrips	Thrips
*Chaetanaphothrips signipennis* *	banana rust thrips	Thrips
*Heliothrips haemorrhoidalis*	greenhouse thrips	Thrips
*Hercinothrips femoralis*	banded greenhouse thrips	Thrips
*Thrips hawaiiensis*	Hawaiian flower thrips	Thrips
*Thrips palmi* *	melon thrips	Thrips
*Thrips tabaci*	onion thrips	Thrips
*Botrytis cinerea* *	gray mold	Fungus
*Fusarium proliferatum* *	*Fusarium* leaf spot	Fungus
Fusarium solani*	*Fusarium* leaf spot	Fungus
*Phyllosticta capitalensis*	*Phyllosticta* leaf spot	Fungus

* Observed in our trial.

**Table 2:** Values are the total numbers of sprays produced (N) by each experimental unit for the length of the trial by variety and treatment.

	TOTAL SPRAYS (N)	
VARIETY	SILICON	CONTROL
**PURPLE** *DENDROBIUM* CV ‘UNIWAI ROYALE’	155 ± 2.00 ^a^	156 ± 2.40 ^a^
**WHITE** *DENDROBIUM* CV ‘UNIWAI MIST’	99 ± 1.46 ^b^	108 ± 1.97 ^b^
**LAVENDER** *DENDROBIUM* CV ‘UNIWAI SUPREME’	49 ± 1.86 ^c^	51 ± 1.20 ^c^

Letters in the same row indicate no significant differences between treatments for each variety, although there were significant differences (α < 0.001) among varieties according to pairwise comparisons between groups using R’s emmeans pairs() function.

## Data Availability

The raw data supporting the conclusions of this article will be made available by the authors on request.

## References

[1] Hara A.H., Hata T.Y., Leonhardt K., Sewake K. (1999). Pests and pest management. Growing Dendrobium Orchids in Hawaii: Production and Management Guide.

[2] USDA NASS (2020). Pacific Region—Hawaii, Hawaii Horticulture and Nursery Products Annual Summary. https://www.nass.usda.gov/Statistics_by_State/Hawaii/Publications/Flowers_and_Nursery_Products/2021%20Hawaii%20Whole%20Flower.pdf.

[3] Loke M. (2018). Farm Disaster Survey Results Kilauea East Rift Zone Eruptions, 2018 Report.

[4] Eng S., Khun T., Esquivel M., Ooki N., Bloese J., Sand S., Lincoln N. (2021). Farmers’ Perceived Needs of Extension’ Support during COVID-19 in Hawaii. J. Ext..

[5] Kirk E., Sand S., Bloese J., Gutierrez-Coarite R., Keach J., Eng S. (2021). COVID-19 Hawaii Agriculture Survey: Initial and On-Going Impacts.

[6] USDA NASS (2020). Floriculture Crops 2019 Summary (December 2020). USDA, National Agricultural Statistics Service. https://www.nass.usda.gov/Publications/Todays_Reports/reports/floran20.pdf.

[7] Sakimura K., Nakahara L.M., Denmark H.A. (1986). A Thrips, Thrips palmi Karny (Thysanoptera: Thripsidae).

[8] Hata T.Y., Hara H.A. (1992). Anthurium thrips, *Chaetanaphothrips orchidii* (Moulton): Biology and insecticidal control on Hawaiian anthuriums. Trop. Pest Manag..

[9] Hata T.Y., Hara A.H., Hu B.K.S., Kaneko R.T., Tenbrink V.L. (1993). Field sprays and insecticidal dips after harvest for pest management of *Frankliniella occidentalis* and *Thrips palmi* (Thysanoptera: Thripidae) on orchids. J. Econ. Entomol..

[10] Waterhouse D.F., Norris K.R. (1989). Chapter 4 *Frankliniella occidentalis* (Pergande). Biological Control Pacific Prospects—Supplement 1.

[11] Johnson M. (1986). Population trends of a newly introduced species, *Thrips palmi* (Thysanoptera: Thripsidae), on commercial watermelon plantings in Hawaii. J. Econ. Entomol..

[12] Gill S., Dutky E., Raupp M., Davidson J., Nakahara S. (2012). Thrips Management in Greenhouses.

[13] Tsai J.H., Yue B., Webb S.E., Funderburk J.E., Hsu H.T. (1995). Effects of Host Plant and Temperature on Growth and Reproduction of *Thrips palmi* (Thysanoptera: Thripidae). Environ. Entomol..

[14] CABI (2023). Thrips palmi. Invasive Species Compendium.

[15] Wang C.L., Chu Y.I. (1986). Rearing method of southern yellow thrips, *Thrips palmi* Karny, in the laboratory. Plant Prot. Bull..

[16] Immaraju J., Paine T.D., Bethke J.A. (1992). Western Flower Thrips (Thysanoptera: Thripidae) Resistance to Insecticides in Coastal California Greenhouses. J. Econ. Entomol..

[17] Mau R.F., Kessing J.L. (1993). Frankliniella occidentalis (Pergande). http://www.extento.hawaii.edu/Kbase/crop/Type/f_occide.htm.

[18] Nagata T., Almeida A.C.L., Resende R.O., Avila A.C., Marullo R., Mound L. (2002). The transmission specificity and efficiency of tospoviruses. Thrips and Tospovirus, Proceedings of the 7th International Symposium on Thysanoptera, Reggio Calabria, Italy, 2–7 July 2001.

[19] Nakahara L.M. (1985). *Thrips palmi* on dendrobium. Proceedings of the 1985 Hawaii Commercial Dendrobium Growers Conference.

[20] Gardener W.D., Leonhardt K.W., Evans D.O., Halloran J.M. (1991). Pest-related flower shipment rejections. Proceedings of the Hawaii Tropical Cut Flower Industry Conference.

[21] Ito J.S., Aragaki M. (1977). Botrytis blossom blight of Dendrobium. Phytopathology.

[22] Srivastava S., Kadooka C., Uchida J.Y. (2018). Fusarium species as pathogen on orchids. Microbiol. Res..

[23] Uchida J.Y., Aragaki M. (1979). Etiology of necrotic flecks on Dendrobium blossoms. Phytopathology.

[24] Seal D.R., Kumar V., Kakkar G., Mello S.C. (2013). Abundance of Adventive *Thrips palmi* (Thysanoptera: Thripidae) Populations in Florida during the First Sixteen Years. Fla. Entomol..

[25] Hata T.Y., Hara A.H., Hansen J.D. (1991). Feeding preference of melon thrips on orchids in Hawaii. HortScience.

[26] Hollingsworth R.G., Hara A.H., Sewake K.T. (2001). Scouting for Thrips in Orchid Plants.

[27] Hollingsworth R.G., Hara A.H., Sewake K.T. (2000). Pesticide use and grower perceptions of pest problems on ornamental crops in Hawaii. J. Ext..

[28] Ancheta D. (2020). Neighbor Islanders Brace for Higher Prices after Young Brothers Gets 46% Rate Increase. Hawaii News Now. https://www.hawaiinewsnow.com/2020/08/17/puc-approved-emergency-percent-rate-increase-young-brothers/.

[29] Reynolds M.G., Keeping J.H., Meyer E.H. (2009). Silicon-augmented resistance of plants to herbivorous insects: A review. Ann. Appl. Biol..

[30] Fateux F., Remus-Borel W., Menzies J.G., Belanger R.R. (2005). Silicon and plant disease resistance against pathogenic fungi. FEMS Microbiol. Lett..

[31] Alhousari F., Greger M. (2018). Silicon and Mechanisms of Plant Resistance to Insect Pests. Plants.

[32] Jones L.H.P., Handreck K.A. (1967). Silica in soils, plants, and animals. Adv. Agron..

[33] Vendrame W.A., Palmateer A.J., Pinares A., Moore K.A., Datnoff L.E. (2010). Silicon fertilization affects growth of hybrid Phalaenopsis orchid liners. Horttechnology.

[34] Mantovani C., Prado R.M., Pivetta K.F.L. (2018). Silicon foliar application on nutrition and growth of *Phalaenopsis* and *Dendrobium* orchids. Sci. Hortic..

[35] Ma J.F., Tamai K., Yamaji N., Mitani N., Konishi S., Katsuhara M., Ishiguro M., Murata Y., Yano M. (2006). A silicon transporter in rice. Nature.

[36] Wagner F. (1940). The importance of silicic acid for the growth of some cultivated plants, their metabolism, and their susceptibility to true mildews. Phytopathol. Zeitschrift..

[37] Samuels A., Glass A., Ehret D., Menzies J. (1991). Distribution of silicon in cucumber leaves during infection by powdery mildew fungus (*Sphaerotheca fuliginea*). Canad. J. Bot..

[38] Rodrigues F.A., McNally D.J., Datnoff L.E., Jones J.B., Labb E.C., Benhamou N., Menzies J.G., Belanger R.R. (2004). Silicon enhances the accumulation of diterpenoid phytoalexins in rice: A potential mechanism for blast resistance. Phytopathology.

[39] Liang Y., Nikolic M., Belanger R.R., Gong H., Song A. (2015). Silicon in Agriculture: From Theory to Practice.

[40] Kvedaras O.L., An M., Choi Y.S., Gurr G.M. (2010). Silicon enhances natural enemy attraction and biological control through induced plant defences. Bull. Entomol. Res..

[41] Hogenhout S.A., Bos J.I.B. (2011). Effector proteins that modulate plant–insect interactions. Curr. Opin. Plant Biol..

[42] Coskun D., Deshmukh R., Sonah H., Menzies J.G., Reynolds O., Ma J.F., Kronzucker H.J., Belanger R.R. (2019). The Controversies of silicon’s role in plant biology. New Phytol..

[43] Cai K., Gao D., Luo S., Zeng R., Yang J., Zhu X. (2008). Physiological and cytological mechanisms of silicon-induced resistance in rice against blast disease. Physiol. Plant..

[44] Gao D., Cai K., Chen J., Luo S., Zeng R., Yang J., Zhu X. (2011). Silicon enhances photochemical efficiency and adjusts mineral nutrient absorption in *Magnaporthe oryzae* infected rice plants. Acta Physiol. Plant..

[45] Resende R.S., Rodrigues F.A., Cavatte P.C., Martins S.C.V., Moreira W.R., Chaves A.R.M., DaMatta F.M. (2012). Leaf gas exchange and oxidative stress in sorghum plants supplied with silicon and infected by Colletotrichum sublineolum. Phytopathology.

[46] Li Y., Bi Y., Ge Y., Sun X., Wang Y. (2009). Antifungal activity of sodium silicate on *Fusarium sulphureum* and its effect on dry rot of potato tubers. J. Food Sci..

[47] Huang C.H., Roberts P.D., Datnoff L.E. (2011). Silicon suppresses *Fusarium* crown and root rot of tomato. J. Phytopathol..

[48] Fortunato A.A., Rodrigues F.A., Baroni J.C.P., Soares G.C.B., Rodriguez M.A.D., Pereira O.L. (2012). Silicon suppresses *Fusarium* wilt development in banana plants. J. Phytopathol..

[49] Almeida G.D., Pratissoli D., Zanuncio J.C., Vicentini V.B., Holtz A.M., Serrão J.E. (2009). Calcium silicate and organic mineral fertilizer increase the resistance of tomato plants to *Frankliniella schultzei*. Phytoparasitica.

[50] Almeida G.D., Pratissoli D., Zanuncio J.C., Vicentini V.B., Holtz A.M., Serrão J.C. (2008). Calcium silicate and organic mineral fertilizer applications reduce phytophagy by *Thrips palmi* Karny (Thysanoptera: Thripidae) on eggplants (*Solanum melongena* L.). Interciencia.

[51] Parthiban P., Chinniah C., Murali Baskaran R.K., Suresh K., Karthick S. (2018). Influence of calcium silicate application on the population of sucking pests of groundnut (*Arachis hypogaea* L.). Silicon.

[52] Dogramaci M., Arthurs S.P., Chen J., Osborne L. (2013). Silicon Applications Have Minimal Effects on *Scirtothrips dorsalis* (Thysanoptera: Thripidae) Populations on Pepper Plant, *Capsicum annum* L.. Fla. Entomol..

[53] Keeping M.G., Miles N., Sewpersad C. (2014). Silicon reduces impact of plant nitrogen in promoting stalk borer (*Eldana saccharina*) but not sugarcane thrips (*Fulmekiola serrata*) infestations in sugarcane. Front. Plant. Sci..

[54] Zellner W. (2020). Personal interview with the author.

[55] Chubachi T., Asano I., Oikawa T. (1986). The diagnosis of nitrogen nutrition of rice plants using chlorophyll meter. Soil Sci. Plant Nutr..

[56] Peris-Felipo F.J., Benavent-Gil Y., Hernandez-Apaolaza L. (2020). Silicon beneficial effects on yield, fruit quality, and shelf-life of strawberries grown in different culture substrates under different iron status. Plant Physiol. Biochem..

[57] R Core Team (2022). R: A Language and Environment for Statistical Computing. R Foundation for Statistical Computing, Vienna, Austria. https://www.R-project.org/.

[58] Posit Team (2023). RStudio: Integrated Development Environment for R. Posit Software, PBC, Boston, MA. http://www.posit.co/.

[59] Knudson C. (2022). glmm: Generalized Linear Mixed Models via Monte Carlo Likelihood Approximation. R Package Version 1.4.4. https://CRAN.R-project.org/package=glmm.

[60] Lenth R. (2023). emmeans: Estimated Marginal Means, aka Least-Squares Means. R Package Version 1.8.6. https://CRAN.R-project.org/package=emmeans.

[61] He J., Khoo G.H., Hew C.S. (1998). Susceptibility of CAM *Dendrobium* leaves and flowers to high light and high temperature under natural tropical conditions. Environ. Exp. Bot..

[62] Kauss H., Seehaus K., Franke R., Gilbert S., Dietrich R.A., Kröger N. (2003). Silica deposition by a strongly cationic proline-rich protein from systemically resistant cucumber plants. Plant J..

